# Spatial Distribution Profiles and Human-Health Risks of Heavy Metals in Surrounding Area Surface Soils of a Petrochemical Complex

**DOI:** 10.3390/ijerph192416930

**Published:** 2022-12-16

**Authors:** Miao Yi, Shiyi Zhang, Min Li, Jun Xiang, Bin Tang, Xiao Yan, Jing Zheng, Guiying Li, Taicheng An

**Affiliations:** 1 Guangdong-Hong Kong-Macao Joint Laboratory for Contaminants Exposure and Health, Guangdong Key Laboratory of Environmental Catalysis and Health Risk Control, Institute of Environmental Health and Pollution Control, Guangdong University of Technology, Guangzhou 510006, China; 2 Guangzhou Key Laboratory of Environmental Catalysis and Pollution Control, Guangdong Technology Research Center for Photocatalytic Technology Integration and Equipment Engineering, School of Environmental Science and Engineering, Guangdong University of Technology, Guangzhou 510006, China; 3 State Environmental Protection Key Laboratory of Environmental Pollution Health Risk Assessment, South China Institute of Environmental Sciences, Ministry of Ecology and Environment, Guangzhou 510530, China

**Keywords:** soil pollution, heavy metals, petrochemical complex, spatial distribution, health risk

## Abstract

Despite the growing concern raised by organic pollutants from the petrochemical industry to the surrounding soils, the heavy metal (HM) pollution in these soils remains understudied. This study investigated the levels, potential sources, and human-health risks of 12 HMs in soils inside and in surrounding areas of a petrochemical complex. Generally, the levels of 12 HMs in all soil samples were lower than the national standard of China, except for the Cd in one surrounding soil sample. Approximately 40.9% and 98.1% of soils around and inside the petrochemical complex, respectively, were at slightly contaminated levels. The HM pollution in 94.4% of soils inside and 32% of soils in surrounding areas were mainly affected by petrochemical production. Human-health risk showed that although As posed an acceptable cancer risk for adults both in and around the complex, high cancer risk for surrounding children from As was observed. Moreover, around the complex, Cr, Cd, and Pb posed acceptable cancer risks for children, while Cd posed an acceptable cancer risk for adults. The spatial distribution of the health risks decreased with increasing distance from the complex. Overall, our results demonstrate that it is essential to minimize human exposure to HMs originating from the petrochemical industry, especially As, Cr, Cd, and Pb.

## 1. Introduction

Contamination of heavy metals (HMs) in soils has becoming a severe environmental problem and global concern because of their ubiquity, mobility, non-degradability, bioaccumulation, and high toxicities [1,2,3]. In China, the National Soil Pollution Survey Bulletin in 2014 revealed that 16.1% of the national total soil exceeded the national standard of China [4]. Among the pollutants in soils, inorganic pollutants were the primary contaminant type, accounting for 82.8% of all the points exceeding the standard [4]. The adverse effects of HMs in the soil on human health are mainly manifested via three exposure pathways: ingestion, dermal contact, and inhalation. These HMs subsequently affect human nervous, cardiovascular, endocrine, immune, reproductive systems and cancer in some cases [2,5,6,7].

HMs in soils can be originated from natural and anthropogenic sources, including transportation, agriculture, and industry [5,6,8,9]. Among these sources, industrial production is considered to be a major source of HMs in soils [10,11,12]. Specifically, the soils in and around the petrochemical industries are frequently contaminated by not only organic pollutants but also HMs [13]. Moreover, HM pollution in soils around petrochemical complexes can occur during oil refining and transportation [6,14]. Solid waste and liquid waste produced during the petroleum refining process also often contain HMs including Cd, Cr, Cu, Ni, Pb, V, and Zn [15,16]. Although the studies on HM pollution in soils of petrochemical complexes [6,15,17] or their surrounding areas [8,13,18,19] are growing, the conclusions from these studies are slightly inconsistent. For instance, the average concentrations of Cu, Zn, Cd, Pb, Cr, As, and Ni in the topsoil of a petrochemical city in Xinjiang Province, China exceeded the background values of them in the soil in Xinjiang [19]. The distribution of Hg, Cd, As, Pb, Cu, and Ni in the surface soil of a decommissioned petrochemical industrial zone in southern China is shown to be mainly affected by petrochemical industrial activities [6]. Moreover, Nadal et al. [15] found that Cr and V in industrial soils inside a petrochemical plant in Spain and Pb in residential soils are significantly higher than those in control soils. In contrast, Zn, Cu, Pb, Cd, Hg, and As in agricultural soil around a petrochemical complex in Guangzhou, China are mainly originated from agricultural production activities and slightly affected by the neighboring petrochemical complex [8]. Despite these advances, only two studies from Serbia have compared HM pollution in soils inside and around petrochemical complexes [20,21]. Relic et al. [20] demonstrate that sediments in the non-petrochemical areas are contaminated with metals originated from petrochemical plants. Their subsequent study showed that the contents of V, Co, Mn, Ni, and As in soils and sediments in the surrounding non-industrial areas near the same petrochemical plant in Serbia are higher than those in the industrial area in 2019 [21]. However, knowledge about pollution levels, spatial distributions and health risks of HMs in soils inside and around petrochemical complexes in China was lacking.

To this end, we selected a petrochemical complex in China as the research object and collected soil samples from the petrochemical complex and its surrounding areas. This petrochemical complex is a large refinery, chemical, and chemical fiber joint enterprise and has operated for approximately 60 years. We aimed to: (1) determine and compare HM concentrations in soils inside and around the petrochemical complex; (2) evaluate soil HM pollution and potential sources in and around the complex; (3) assess the health risks of humans exposed to these HM-contaminated soils; and (4) map the spatial distribution of HM contents and health risks.

## 2. Materials and Methods

### 2.1. Study Area and Sample Collection

**Figure 1:**
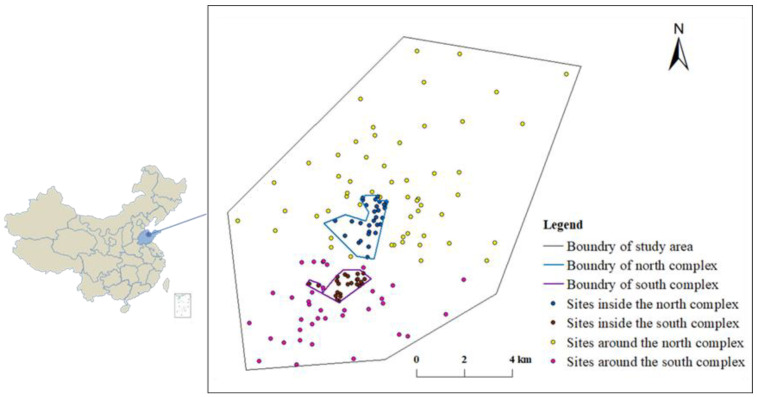
Soil sampling sites of the study area.

The study region covers an area of 16.65 km^2^, characterized by humid monsoon climate and distinct seasonality, with an annual average temperature of 16–18 °C and annual average precipitation of ~700 mm. The south and southwest winds are the dominant wind directions, followed by the north to northeast winds. The petrochemical complex has a comprehensive refining process, with a processing capacity of 13 million tons per year, and its ethylene production capacity mounts up to 800,000 tons per year. The complex mainly produces gasoline, diesel, asphalt, polyethylene, polypropylene, polyvinyl chloride, and other petrochemical products. In this study, the petrochemical complex is divided into two areas based on the origin of the crude oil used: (1) the southern zone (domestic crude oil) and (2) the northern zone (imported crude oil). Further, this study area is divided into two main parts: the area inside the complex and the area outside the complex (Table S1). The soil samples were collected according to the Technical Specification for Soil Environmental Monitoring of China (HJ/T 166-2004). The distribution of sampling points is shown in Figure 1. In terms of the grid distribution method, 37 topsoil samples (0–20 cm) within 4 km around the south complex, and 56 topsoil samples within 10 km around the north complex were collected. In total, 53 topsoil samples were collected within the complex, with 24 and 29 topsoil samples inside the south and north complex, respectively (Table S1). All the soil samples were stored in polyethylene sealable bags at −20 °C for further analysis.

### 2.2. Chemical Analysis

The soil samples were processed according to the methods described in the Environmental Protection Standards of the People’s Republic of China (HJ 803-2016). The debris, such as sticks, leaves, and stones, was removed from the soil samples. The soil samples were subsequently air-dried, and roughly-ground and finely-ground to pass a sieve with a pore size of 0.15 mm (100 mesh). Then, 0.1 g of the sample was placed in a closed Teflon digestion tank, 4.5 mL hydrochloric acid and 1.5 mL nitric acid were added, and the samples were digested with a microwave digestion instrument (MARS-6, CEM). The concentrations of V, Cr, Mn, Co, Ni, Cu, Zn, As, Cd, Sb, and Pb were determined using inductively coupled plasma mass spectrometry (7900 ICP-MS, Agilent). Furthermore, Hg was analyzed using thermal decomposition, amalgamation, and atomic absorption spectrophotometry (Hydra-C, Leeman).

### 2.3. Quality Assurance and Quality Control

The Chinese standardized reference material (GSS-5) was used to ensure quality assurance/quality control. Two laboratory blank samples were prepared for each batch of samples. Indicatively, the results were all below the lower limit of determination. The recoveries of 12 HMs ranged from 69–127% (Figure S1).

### 2.4. Data Analysis

#### 2.4.1. Geoaccumulation Index

The geoaccumulation index (*I_geo_*) was initially proposed by Muller [22] and has been applied to assess soil contamination of HMs [23]. It can be calculated using Equation (1):

(1) $$\begin{array}{c} I_{\mathrm{geo}}{=\log}_{2}\left(\frac{C_{i}}{1{.5B}_{i}}\right) \end{array}$$

where *C_i_* is the total concentration of element *i* in soil sample (mg/kg); *B_i_* is the geochemical background content of element *i* (mg/kg); the correction index is 1.5, which is typically used to characterize sedimentary characteristics, rock geology, and detect very small anthropogenic influences [24,25]. In this study, *B_i_* is the background content of element *i* in soils of the study area [26]. According to Muller [22], *I_geo_* can be divided into seven grades, as described in Table S2.

#### 2.4.2. Nemerow Integrated Pollution Index

The Nemerow integrated pollution index (*NPI*) [27] is a comprehensive pollution index which can be used to assess overall pollution level of HMs [28]. The calculation formulae for single pollution index (*PI*) and *NPI* are shown in Equations (2) and (3), respectively:

(2) $$\begin{array}{c} \mathrm{PI}=\frac{C_{i}}{S_{i}} \end{array}$$

(3) $$\begin{array}{c} \mathrm{NPI}=\sqrt{\frac{{\mathrm{PI}}_{\mathrm{imax}}^{2}{+\mathrm{PI}}_{\mathrm{iave}}^{2}}{2}}\; \end{array}$$

where *C_i_* is the total concentration of element *i* in the soil samples (mg/kg); *S_i_* is the reference standard of element *i* (mg/kg); *PI_iave_* is the average single pollution index of all HMs; *PI_imax_* is the maximum single pollution index of all HMs [29,30]. In this study, *S_i_* is the background content of element *i* in soils of the study area [26]. *PI* and *NPI* are divided into five grades, as described in Table S3.

#### 2.4.3. Health Risk Assessment

The human health risk method proposed by U.S. Environmental Protection Agency (USEPA) [31] was used to assess the carcinogenic and non-carcinogenic risks of people exposed to HMs. The calculation of exposure in this model is mainly divided into three categories: oral ingestion of soil, dermal contact with soil, and inhalation of soil particulate matter. All these exposures were estimated using Equations (4)–(8):

(4) $$\begin{array}{c} {\mathrm{ADD}}_{\mathrm{ing}}=\frac{C_{\mathrm{soil}}\;\times \;\mathrm{IngR}\;\times \;\mathrm{EF}\;\times \;\mathrm{ED}}{\mathrm{BW}\;\times \;\mathrm{AT}}{\;\times \;10}^{-6} \end{array}$$

(5) $$\begin{array}{c} {\mathrm{ADD}}_{\mathrm{inh}}=\frac{C_{\mathrm{soil}}\;\times \;\mathrm{InhR}\;\times \;\mathrm{EF}\;\times \;\mathrm{ED}}{\mathrm{PEF}\;\times \;\mathrm{BW}\;\times \;\mathrm{AT}}\; \end{array}$$

(6) $$\begin{array}{c} {\mathrm{ADD}}_{\mathrm{der}}=\frac{C_{\mathrm{soil}}\;\times \;\mathrm{SA}\;\times \;\mathrm{AF}\;\times \;\mathrm{ABF}\;\times \;\mathrm{EF}\;\times \;\mathrm{ED}}{\mathrm{BW}\;\times \;\mathrm{AT}}{\;\times \;10}^{-6} \end{array}$$

(7) $$\begin{array}{c} \mathrm{HI}=\sum^{\;}{\mathrm{HQ}}_{i}=\sum^{\;}\frac{{\mathrm{ADD}}_{i}}{{\mathrm{RfD}}_{i}} \end{array}$$

(8) $$\begin{array}{c} \mathrm{TCR}=\sum^{\;}{\mathrm{CR}}_{i}=\sum^{\;}{\mathrm{ADD}}_{i}{\;\times \;\mathrm{SF}}_{i} \end{array}.$$

where *C_soil_* is the total concentration of heavy metal in soil sample (mg/kg); *ADD_ing_* is the average daily dose of the oral ingestion of soils (mg/kg day); *ADD_inh_* is the average daily dose of the inhalation of soils (mg/kg day); *ADD_der_* is the average daily dose of the dermal contact with soils (mg/kg day); and *HQ* is the hazard quotient, *HI* is the hazard index, *CR* is the carcinogenic risk, and *TCR* is total carcinogenic risk. Note that other parameters are listed in Table S4, while the values of the reference dose [*RfD*, mg/(kg·d)] and slope factors [*SF*, (kg·d)/mg] are summarized in Table S5.

According to the health risk assessment model [32], *HI* > 1 indicates non-carcinogenic health risk, *TCR* > 1 × 10^−4^ indicates a high risk of developing cancer for humans, *TCR* of 1 × 10^−4^ – 1 × 10^−6^ indicates an acceptable risk of cancer, and *TCR* < 1 × 10^−6^ indicates a negligible cancer risk.

#### 2.4.4. Statistical Analysis

The data calculations and analyses were conducted using the SPSS version 26.0 (IBM Corporation, New York, NY, USA). The diagrams were constructed using Origin 2022b software (OriginLab Corporation, Northampton, MA, USA). Spatial distribution maps of the HMs were composed using ArcMap 10.8 (Environment System Research Institute Inc, RedLands, CA, USA). All the data were tested for normality. As the data did not conform to a normal distribution, non-parametric tests were used in subsequent data testing and analysis, and the median value was applied as an indicator for the discussion in next sections. SPSS was used to screen outliers, which were removed from the statistical analysis in this study.

## 3. Results and Discussion

### 3.1. HM Concentrations Inside and Around the Petrochemical Complex

Table 1 summarizes the descriptive statistics of HM concentrations in the 53 and 93 surface soil samples collected from the study area inside and around the petrochemical complex, respectively. The data of HMs in soil samples in different countries and regions are listed in Table S6. In this study, in the area around the petrochemical complex, the median values of 12 HMs in soil samples were as follows: Mn (443 mg/kg) > Zn (68.3 mg/kg) > V (51.8 mg/kg) > Cr (39.7 mg/kg) > Ni (26.8 mg/kg) > Cu (22.1 mg/kg) > Pb (16.7 mg/kg) > As (9.73 mg/kg) > Co (9.51 mg/kg) > Sb (0.79 mg/kg) > Cd (0.12 mg/kg) > Hg (0.019 mg/kg). Figure S2 reveals that there were 39.9% of As, 35.8% of Zn, 34.2% of Sb, and 29.1% of Cd around the complex, which were exceeded the background values [26]. In particular, one soil sample around the petrochemical complex exhibited high Cd concentration (0.32 mg/kg), exceeding the risk screening value (0.3 mg/kg) of the Risk Control Standard for Soil Contamination of Agricultural Land (GB 15618-2018) issued by the Ministry of Ecology and Environment of China. This site is approximately 3 km away from the eastern boundary of the south petrochemical complex, located in a corner near a residential house, where domestic waste is stacked. As reported, domestic waste, such as plastic packing bags could also generate HMs [33]. However, the HM concentrations in soils around the complex were generally higher than those in the residential area in Spain [15], agricultural soils in northeastern Iran [34], Tarkwa [35] and natural soils in Pearl River Delta, China [36]. What is more, the concentrations of Cr, Ni, Cu, Zn, As, and Pb in soils around the complex were significantly higher than those in agricultural soils in other area of the same city [37]. These indicated that the petrochemical complex potentially led to HM pollution in its surrounding areas.

With respect to the soils inside the petrochemical complex, the median values of HMs in soils were in order of Mn (494 mg/kg) > Zn (88.4 mg/kg) > V (63.9 mg/kg) > Cr (45.6 mg/kg) > Ni (28.4 mg/kg) > Pb (19.5 mg/kg) > Cu (18.7 mg/kg) > As (14.1 mg/kg) > Co (9.77 mg/kg) > Sb (0.75 mg/kg) > Cd (0.072 mg/kg) > Hg (0.016 mg/kg). The HM contents in all soil samples inside the complex were substantially lower than the risk screening values of Category II of Land Use in the Risk Control Standard for Soil Contamination of Development Land in China (GB 36600-2018). However, it is worth noting that there still were 23.6% of Sb, 30.7% of Pb, 75.7% of Zn, and 100% of As inside the complex exceeded the background values (Figure S3). The concentrations of V, Cr, Mn, Co, Zn, As, and Pb inside complex were significantly higher than those around complex (*p* < 0.05). In particular, the average level of Zn inside the complex was nearly twice as much as those in the surrounding areas. In contrast, the concentrations of Cu, Cd, and Hg around the complex were markedly higher than those inside complex (Table 1, *p* < 0.05), which is possibly due to traffic and agricultural activities [38,39]. Indicatively, the concentrations of As, Sb, and Hg in southern complex were significantly higher than those in the northern complex (Table S7, *p* < 0.05), which were potentially attributed to the differences in the crude oil used in the north and south complexes. Specifically, the south complex uses domestic crude oil, characterized by higher sulfur and acid contents than imported crude oil used by north complex. These resulted in a higher content of SOx emitted from south complex than north complex, leading to subsequent HM pollution of soils in south complex [19,40,41]. As whole, soil contaminations of V, Cr, Mn, Co, Zn, As, and Pb inside the complex were significantly heavier than those around the complex, which could pollute the surrounding environment by oil leakage, discharge of industrial waste and residue atmospheric deposition of emissions from industrial production [3,6,19].

The median value of Zn (88.4 mg/kg) in soils inside petrochemical complex in our study exceeded the background concentration (76.4 mg/kg) of the same city [26]. In particular, As concentrations at all sampling points in the complex exceeded the background value (10.0 mg/kg) and was nearly twice as much in the petrochemical complex in Serbia [21] and Spain [15]. Moreover, the median concentration of Hg (0.016 mg/kg) inside the complex in this study was order of magnitude lower than that in the petrochemical complex of Guangdong Province (0.18 mg/kg) [6].

### 3.2. HM Pollution Inside and Around the Petrochemical Complex

**Figure 2:**
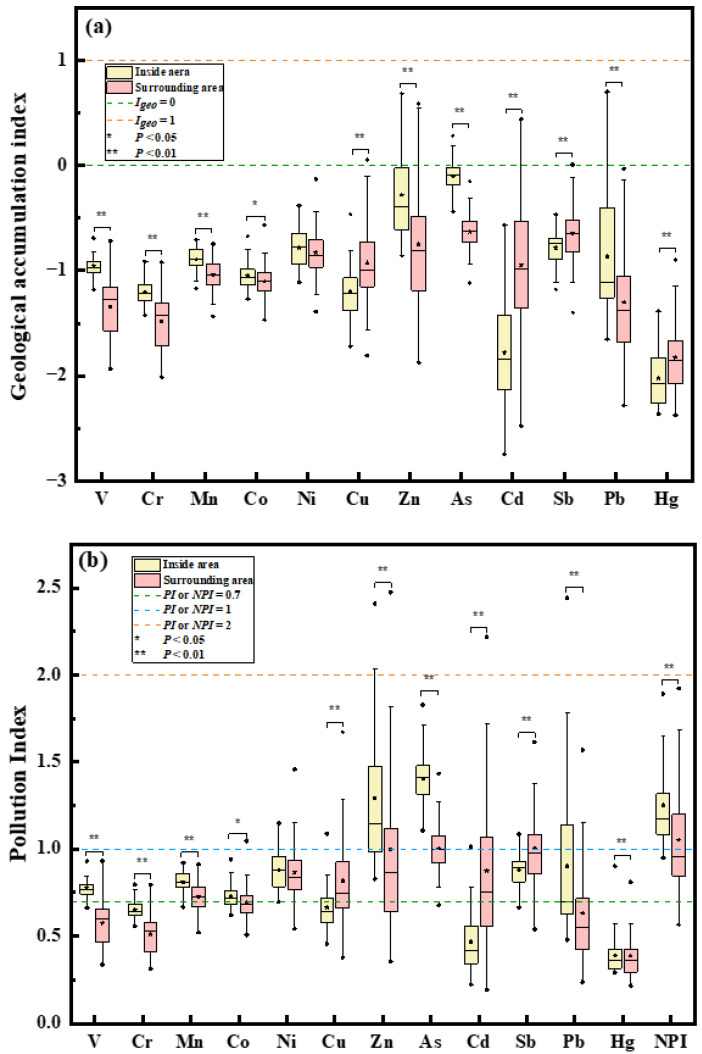
The Geological accumulation indices (**a**) and the Pollution indices (**b**) of HMs inside and around the petrochemical complex.

To further assess the overall pollution levels of HMs in soils, we calculated the values of *I_geo_* and *PI* [1,30]. Figure 2a reveals the following order of the median values of the *I_geo_* of the HMs inside the petrochemical complex: As > Zn > Sb > Ni > Pb > Mn > V > Co > Cu > Cr > Cd > Hg. Around the complex, the order of *I_geo_* was as follows: As > Sb > Zn > Ni > Cu > Cd > Mn > Co > Pb > V > Cr > Hg. Apart from Cu, Ni, Cd, Sb, and Hg, the *I_geo_* values of the eight HMs inside the complex were significantly higher than those around the complex (*p* < 0.05). The *I_geo_* values of 22.92% of Zn, 20.75% of As, and 7.69% of Pb in soils inside the complex were within uncontaminated-to-moderately contaminated range (Table S8). Furthermore, around the complex, the *I_geo_* values of 14.77% of Zn, 8.7% of Cd, 1.12% of Cu, and 1.1% of Sb revealed uncontaminated-to-moderately contaminated conditions (Table S8). The *I_geo_* was calculated by local background values and correction index used to analyze natural fluctuations and underlying anthropogenic influences [29], which could be used to determine the two sources of HMs: high value of *I_geo_* indicating obvious anthropogenic source [42]. Based on the results mentioned above (at uncontaminated-to-moderately contaminated level around the complex), HMs around the complex were not only from natural sources, but also anthropogenic sources, such as petrochemical production [42].

Meanwhile, the *PI*, which is the ratio of the actual concentrations of HMs in soils to the background values of soils, was calculated to assess HM contamination inside and around the petrochemical complex (Figure 2b and Table S9). The *PI* values of Cu, Cd, and Hg around the complex were higher than those inside the complex, while the *PI* indices of other HMs (except Ni) inside the complex were higher than those around the complex (*p* < 0.05). These results were well in agreement with the values of *I_geo_*. It is noteworthy that HM contamination around the complex should raise our attention, for nearly or more than half of Mn, Co, Ni, Cu, As, and Sb in soils around the complex were at the precaution level; in particular, more than 20% of the soils in surrounding areas were slightly contaminated with Zn, As, Cd, and Sb (Table S9). Furthermore, approximately 99% of As in soils around the complex had the *PI* values at the level of precaution and slightly contaminated; what is more, all the soil samples inside the complex were slightly contaminated with As, indicating that soil As both inside and around the complex was strongly affected by the petrochemical production activities.

Additionally, owing to the fact that HM pollution in soils is usually caused by multiple HMs at the same time [43] and the comprehensive pollution level is a non-negligible factor in pollution assessment, we further calculated the *NPI* values. As Figure 2b shows, *NPI* values ranged from 0.95 to 1.89 with a median value of 1.71 inside the petrochemical complex; while the median *NPI* values ranged from 0.6 to 1.78 with a median value of 0.93 around the complex. Obviously, the *NPI* values in the complex were higher than those around the complex (*p* < 0.05), indicating that the overall pollution level of HMs in soils of the complex was higher than that of the surrounding areas. Furthermore, approximately 40.86% of soils around the complex had *NPI* values ranging from 1 to 2, which were at the slightly polluted level [44]. What is more, the comprehensive pollution level of soils around the complex was heavier than that in agricultural soils in Dongguan [45] and Hunchun basin in China [46]. In addition, it is of concern that almost all soil samples in the petrochemical complex were at the slightly contaminated level, although the comprehensive pollution level was relatively lower than that in soil inside some smelters [30,47].

### 3.3. Source Analysis of HMs

Correlation analysis is usually used to evaluate the relevant level of the metals and determine whether they have homology [19]. Spearman’s correlation matrix is presented in Table S10. The correlation coefficients among V, Cr, Mn, Co, and As, as well as Cu-Cd-Sb, Cr-Zn, Mn-Ni, Co-Ni, Sb-Ni, Zn-Pb, and Cd-Hg ranged from 0.51 to 0.89, which showed high significant positive correlations (*p* < 0.01). These results suggest that the sources of these HMs are likely to be similar. Similarly, Relic et al. [20] demonstrate that Co, Cr, Ni, Mn and V in the sediments sampled from the petrochemical complex show the significant correlations with each other. Otherwise, the concentrations of V-Cu, V-Cd, Cu-As, and Cd-As presented significant negative correlations (*p* < 0.01), indicating different sources of these HMs.

**Figure 3:**
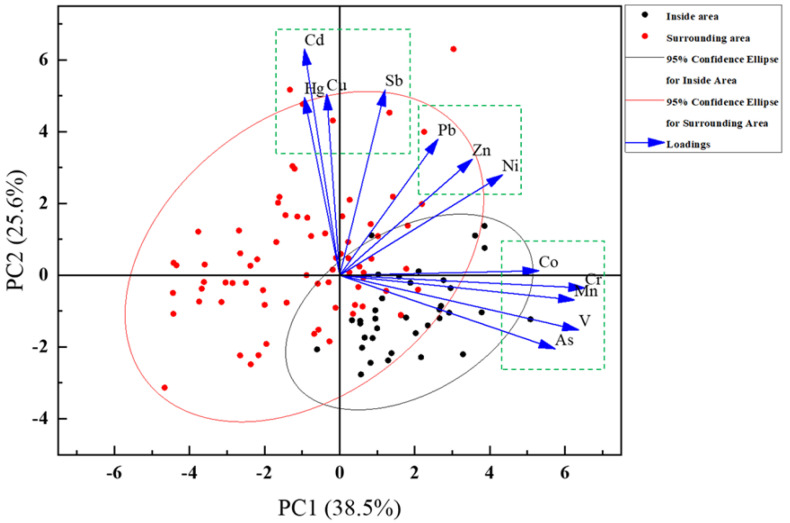
Principal component analysis of the HMs inside and around the petrochemical complex.

Principal Component Analysis (PCA) was performed to give assistance to further identify the potential sources of the 12 HMs inside and around the petrochemical complex area. Three principal components were obtained with eigenvalues > 1, accounting for 38.5%, 25.6%, and 11.4%. The first principal component (PC1) and second principal component (PC2) were considered in this study, which together accounted for 64.1% of the total variance. The score plot is shown in Figure 3: the sampling sites inside the complex were clearly separated from those around the complex and mainly scattered in the first quadrant, indicating that PC1 mostly represented the petrochemical pollution source, while PC2 represented other sources. Furthermore, 94.4% of soils inside the complex and 32% of soils in surrounding areas were primarily affected by PC1, indicating that they were mainly influenced by petrochemical production activities.

Moreover, the data of HM concentrations appeared to be clustered into three different groups. In Group 1, V, Cr, Mn, Co, and As agglomerated into the group inside the petrochemical complex, which appeared to provide further evidence that the petrochemical production activities as the predominant source. In Group 2, Cu, Cd, Sb, and Hg formed one group, which was primarily derived from other sources, such as transportation and agriculture and showed significant higher levels around the complex than those inside the complex. The above results of the Spearman correlation analysis (Table S9) well supported the conclusions of the principal component analysis.

The sites around the petrochemical complex were mostly identified close to roads and agricultural land. As reported, Cu and Cd are the elements of pesticides and fertilizers used in agricultural activities [38,48,49]. In addition, Cd is commonly used as an electroplating layer in automobile manufacturing to protect automotive materials from corrosion [50,51,52], and Hg is emitted from the combustion of gasoline and diesel in automotive vehicles [39]. Furthermore, Manno et al. [53] and Bosco et al. [18] reported that traffic can lead to Sb pollution. Overall, our findings indicate that PC2 represents traffic and agricultural production activities. In addition, after the field visits, several plastics, rubber, and other chemical plants are located around the complex. Previous studies have indicatively illustrated that rubber contains HMs [54], and plastics can release HMs into the environment, thereby inducing a corresponding HM pollution in the neighboring soils [55]. These factories around the petrochemical complex were among the sources of HMs represented by PC2.

Particularly, Zn, Pb, and Ni clustered in Group 3, which was identified in the middle of the Groups 1 and 2. This finding clearly suggests their multiple sources, including petrochemical production activities, traffic, and agricultural production activities. It was considered by previous studies that Pb is originated from petroleum smelting and transportation as well as road traffic [39,56]. The wear of car tires results in the release of Zn, but the wear of asphalt pavement contributes to Ni pollution [39]. In addition, Ni is considered as the main pollutant in petrochemical plants [6,57].

### 3.4. Human Health Risks

Health risks for adults and children were assessed separately based on the dose-response model recommended by the USEPA [32]. In the present study, 12 HMs were considered in the non-carcinogenic risk assessment, while analyzing the carcinogenic risks, 6 HMs including Cr, Co, Ni, As, Cd, and Pb were assessed [3,25,58,59]. The median HI and TCR values are summarized in Table 2. Inside the petrochemical complex, the median HI values of the 12 HMs followed the order: As (7.69 × 10^−2^) > Cr (3.01 × 10^−2^) > Mn (2.50 × 10^−2^) > V (2.10 × 10^−2^) > Pb (9.24 × 10^−3^) > Sb (3.59 × 10^−3^) > Ni (2.31 × 10^−3^) > Co (8.53 × 10^−4^) > Cu (7.64 × 10^−4^) > Zn (4.81 × 10^−4^) > Cd (1.57 × 10^−4^) > Hg (9.18 × 10^−5^). What’s more, the median HI values of all HMs for adults around the complex were in order of As (5.30 × 10^−2^) > Cr (2.61 × 10^−2^) > Mn (2.26 × 10^−2^) > V (1.70 × 10^−2^) > Pb (7.68 × 10^−3^) > Sb (3.84 × 10^−3^) > Ni (2.18 × 10^−3^) > Cu (8.92 × 10^−4^) > Co (8.30 × 10^−4^) > Zn (3.59 × 10^−4^) > Cd (2.85 × 10^−4^) > Hg (1.07 × 10^−4^). Comparatively, the median TCR values of all HMs for children around the complex also followed the law of above. For both adults and children around the complex, the HI values of the 12 HMs were all < 1, indicating that none of the HMs in soils posed non-carcinogenic health risks to them. Furthermore, the HI values of the 12 HMs for adults inside the complex also indicate negligible non-carcinogenic health risks.

In terms of carcinogenic health risks for adults inside the petrochemical complex, the median values of six HMs were listed in the following order: As (3.46 × 10^−5^) > Cr (9.50 × 10^−7^) > Cd (6.68 × 10^−7^) > Pb (2.67 × 10^−7^) > Co (1.65 × 10^−8^) > Ni (4.07 × 10^−9^). As for the carcinogenic risks around the petrochemical complex, the median TCR values of the six HMs for adults were as follows: As (2.38 × 10^−5^) > Cd (1.21 × 10^−6^) > Cr (8.26 × 10^−7^) > Pb (2.89 × 10^−7^) > Co (1.61 × 10^−8^) > Ni (3.85 × 10^−9^), and the same pattern was obtained for children. Additionally, the TCR values of Cr, Co, Ni, and As inside the complex were significantly higher than those in surrounding areas, while Cu showed the opposite pattern (*p* < 0.05). Furthermore, As for adults both inside and around the complex, Cr, Cd, and Pb for children around the complex were estimated to be at acceptable carcinogenic risk level. The cancer risks of Cd for adults around the complex were within an acceptable range. In particular, the median TCR value of As for children around the complex exceeded 1 × 10^−4^, signifying a strong risk of developing cancer. Notably, this finding was in line with that of previous studies, which have reported that people are more likely to be exposed to As from soil [3,60,61].

Compared to the results of Wang et al. [6], the total HI and TCR values were relatively high in this study both inside and around the petrochemical complex. Furthermore, the total HI and TCR values inside the complex for adults were higher than those around the complex, indicating higher health risks for adults inside the complex than those around the complex. Additionally, the present study revealed that the carcinogenic and non-carcinogenic risks were higher for children than adults (*p* < 0.05), possibly because children simply spend more time outdoors [62], resulting in higher frequency of children’s exposure to soil and higher ingestion rates than by adults [3].

### 3.5. Spatial Distribution of HM Contents and Health Risks

The estimated concentration maps for V, Mn, Cr, As, Cu, Cd, Hg, Zn, Co, Ni, Sb, and Pb are shown in Figure S4. The spatial distributions of the concentrations of V, Mn, Cr, and As in soils were similar and decreased as follows: inside the complex > around the complex boundary > away from the complex boundary, indicating that the petrochemical industry production activities strongly affected the HM levels in the soils near the petrochemical complex. This conclusion is consistent with our result of PCA. Similarly, a study demonstrates that the contents of Cr and Mn are relatively high in refinery sludge, indicating a primary HM source of soils inside the petrochemical industry [63]. In general, given the presence of V in crude oil, the V may release into the environment from the refineries, thereby polluting the soil inside the complex [13]. Moreover, Zn was mainly distributed in the southern complex, which might potentially be bolstered by the SOx emitted from the use of high-sulfur crude oil in the southern complex [19]. Additionally, the hot sites in the southeast and southwest corners were identified around the south complex of V and Mn, respectively. They were all located along the roadside in the residential area, which may have been affected by traffic emissions and the combustion of fossil fuels [15].

**Figure 4:**
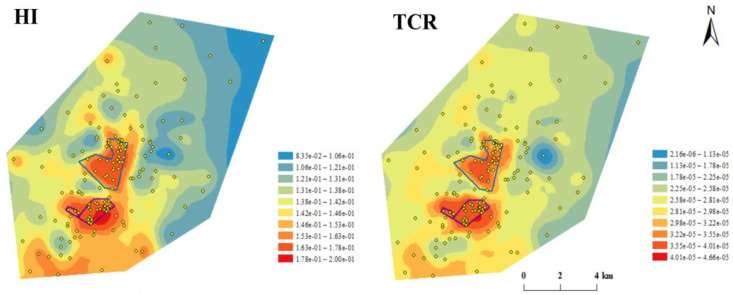
The spatial distribution of *HI* and *TCR* for adults.

Compared with the spatial distribution of HM contents, the spatial distribution of human-health risks is more worthy of attention. The estimated level maps for *HI* and *TCR* of all HMs are shown in Figure 4. *HI* and *TCR* showed similar patterns in spatial distribution. Both carcinogenic and non-carcinogenic risks showed higher levels in the petrochemical complex than those in the surrounding areas. With the increase of the distance from the complex, both carcinogenic and the non-carcinogenic risks showed downward trends, indicating that the health risks in the study area mainly attributed to the petrochemical complex. Particularly, due to the impact of petrochemical downstream enterprises, higher carcinogenic and non-carcinogenic risks were also observed in the southeast part of the surrounding area, which is consistent with the relatively high concentration of all HMs in this area in the estimated concentration maps.

## 4. Conclusions

In summary, we investigated and compared the pollution characteristics, spatial distributions, potential sources, and health risks of 12 HMs in surface soils of a petrochemical complex and its surrounding areas. Overall, the petrochemical complex exacerbated the HM pollution of soils not only inside but also around the complex, thereby triggering health risks to the workers and surrounding residents. With the increase of the distance from the complex, both the carcinogenic and the non-carcinogenic risks showed downward trends. Particularly, the health risk assessment demonstrated that As in soils posed an acceptable carcinogenic risk for adults both inside and around the complex as well as a high carcinogenic risk for children around the complex. Most notably, the actual health risks might be underestimated in this study because of the potential synergy effects among different HMs.

Therefore, while paying attention to the organic pollution of the petrochemical industry, the pollution of HMs cannot be neglected. What is more, the hotspot areas with high HM concentrations and high health risks identified by spatial distribution need to attract our attention and take corresponding measures. Accordingly, it is necessary to take measures to improve the local soil quality, especially from As, Cr, Cd, and Pb pollution, thereby minimizing the health risks of the population. However, this study only used the model to evaluate the possible health risks of HM pollution to adults and children in the study area but did not figure out the exact health impacts to human beings. Consequently, further research focused on the health effects of soil pollution in the petrochemical industry on occupational workers and the surrounding population is highly recommended in the future.

## Figures and Tables

**Table 1:** Descriptive statistics of HM contents (mg/kg) in soils inside (*n* = 53) and around (*n* = 93) the petrochemical complex.

HMs	Inside Area					Surrounding Area					Background Values [26]
	Min	Max	Median	Mean	SD	Min	Max	Median	Mean	SD	
V	55.2	77.4	63.9	64.7	4.46	32.7	75.9	51.8	50.0	8.80	83.2
Cr	39.5	95.8	45.6	47.1	7.59	26.3	56.1	39.7	38.6	6.54	70.8
Mn	404	619	494	495	42.2	336	542	443	443	45.4	605
Co	8.47	12.8	9.77	9.94	0.87	7.38	13.8	9.51	9.55	0.10	13.6
Ni	22.2	103	28.4	31.2	13.0	18.3	117	26.8	28.9	11.0	32.0
Cu	13.8	34.7	18.7	19.5	4.50	12.4	56.1	22.1	24.7	8.41	28.9
Zn	63.2	4500	88.4	207	604	31.3	453	68.3	84.4	57.7	76.4
As	11.1	18.3	14.1	14.1	1.47	6.92	15.1	9.73	9.82	1.24	10.0
Cd	0.036	0.97	0.072	0.10	0.13	0.044	0.58	0.12	0.15	0.079	0.16
Sb	0.54	2.45	0.75	0.84	0.34	0.47	2.17	0.79	0.83	0.23	0.82
Pb	13.3	81.1	19.5	26.2	14.2	8.60	87.1	16.7	20.2	12.4	27.9
Hg	0.013	0.043	0.016	0.019	0.007	0.013	0.054	0.019	0.021	0.008	0.045

**Table 2:** Non-carcinogenic (HI) and carcinogenic health risks (TCR) of HMs in soils.

Risk	HMs	Inside Area	Surrounding Area	
		Adults	Adults	Children
HI	V	2.10 × 10^−2^	1.70 × 10^−2^	9.54 × 10^−2^
	Cr	3.01 × 10^−2^	2.61 × 10^−2^	1.54 × 10^−1^
	Mn	2.50 × 10^−2^	2.26 × 10^−2^	1.15 × 10^−1^
	Co	8.53 × 10^−4^	8.30 × 10^−4^	5.07× 10^−3^
	Ni	2.31 × 10^−3^	2.18 × 10^−3^	1.40 × 10^−2^
	Cu	7.64 × 10^−4^	8.92 × 10^−4^	5.71 × 10^−3^
	Zn	4.81 × 10^−4^	3.59 × 10^−4^	2.29 × 10^−3^
	As	7.69 × 10^−2^	5.30 × 10^−2^	3.40 × 10^−1^
	Cd	1.57 × 10^−4^	2.85 × 10^−4^	1.59 × 10^−3^
	Sb	3.59 × 10^−3^	3.84 × 10^−3^	2.27 × 10^−2^
	Pb	9.24 × 10^−3^	7.68 × 10^−3^	4.87 × 10^−2^
	Hg	9.18 × 10^−5^	1.07 × 10^−4^	6.72 × 10^−4^
	Total	1.70 × 10^−1^	1.35 × 10^−1^	8.05 × 10^−1^
TCR	Cr	9.50 × 10^−7^	8.26 × 10^−7^	3.98 × 10^−6^
	Co	1.65 × 10^−8^	1.61 × 10^−8^	2.68 × 10^−8^
	Ni	4.07 × 10^−9^	3.85 × 10^−9^	6.41 × 10^−9^
	As	3.46 × 10^−5^	2.38 × 10^−5^	1.53 × 10^−4^
	Cd	6.68 × 10^−7^	1.21 × 10^−6^	7.82 × 10^−6^
	Pb	2.67 × 10^−7^	2.89 × 10^−7^	1.43 × 10^−6^
	Total	3.65 × 10^−5^	2.62 × 10^−5^	1.66 × 10^−4^

## Data Availability

Not applicable.

## Supplementary Materials

The following supporting information can be downloaded at: https://www.mdpi.com/article/10.3390/ijerph192416930/s1. Figure S1: recovery rate of HMs in soil samples; Figure S2: cumulative probability curves of the contents of Zn, As, Cd, and Sb in soils around the complex; Figure S3: cumulative probability curves of the contents of Zn, As, Sb, and Pb in soils inside the complex; Figure S4: estimated Ordinary Kriging concentration maps for V, Mn, Cr, As, Cu, Cd, Hg, Zn, Co, Ni, Sb, and Pb (mg/kg); Table S1: soil samples collection information; Table S2: the classification of geoaccumulation index (*I_geo_*); Table S3. the classification of Pollution index (*PI*) and Nemerow integrated pollution index (*NPI*); Table S4: parameters of human-health risk assessment models; Table S5: summary of reference dose (*RfD*) and slope factor (*SF*) of HMs; Table S6: concentrations (mg/kg) of HMs in soil samples in different studies; Table S7: descriptive statistics of HM contents (mg/kg) in soils of north complex (*n* = 24) and south complex (*n* = 29); Table S8: the proportion of different soil grades of HMs inside and around the petrochemical complex classified by *I_geo_*; Table S9: the proportion of different soil grades of HMs inside and around the petrochemical complex classified by *PI* and *NPI;* Table S10: correlations between twelve HM concentrations in soils [3,6,8,15,19,21,31,32,34,35,36,48,58,59,64,65].
