# Supplementary material for: Spatial Distribution Profiles and Human-Health Risks of Heavy Metals in Surrounding Area Surface Soils of a Petrochemical Complex
Source: Int J Environ Res Public Health. 2022 Dec 16;19(24):16930. doi: 10.3390/ijerph192416930 (PMC9778647; doi:10.3390/ijerph192416930)
Supplement: Supplementary file 1 [file ijerph-19-16930-s001.zip › ijerph-2060538-supplementary.pdf]

International Journal of
Environmental Research and Public Health

MDPI

Supplementary Materials

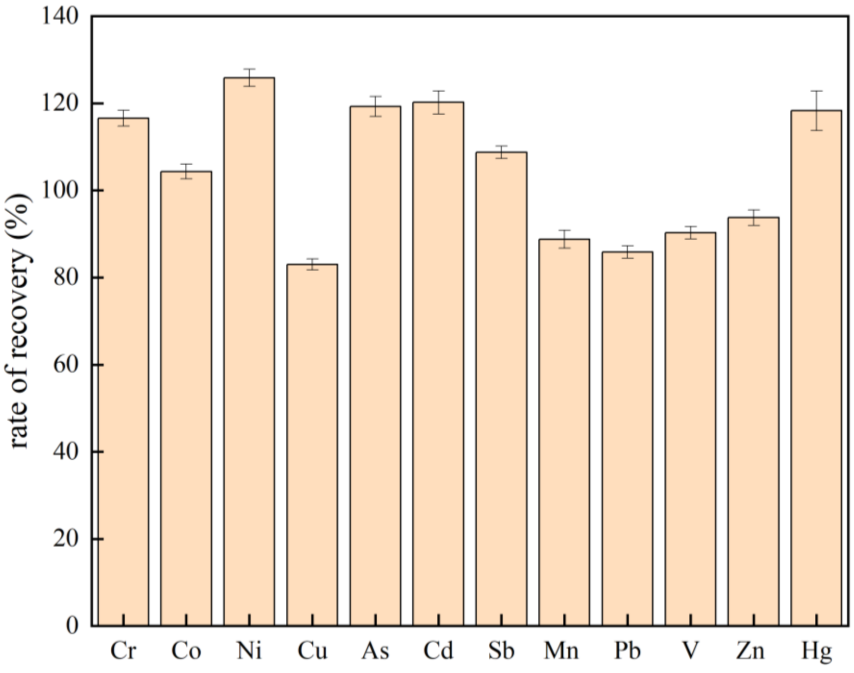

Figure S1. Recovery rate of HMs in soil samples.

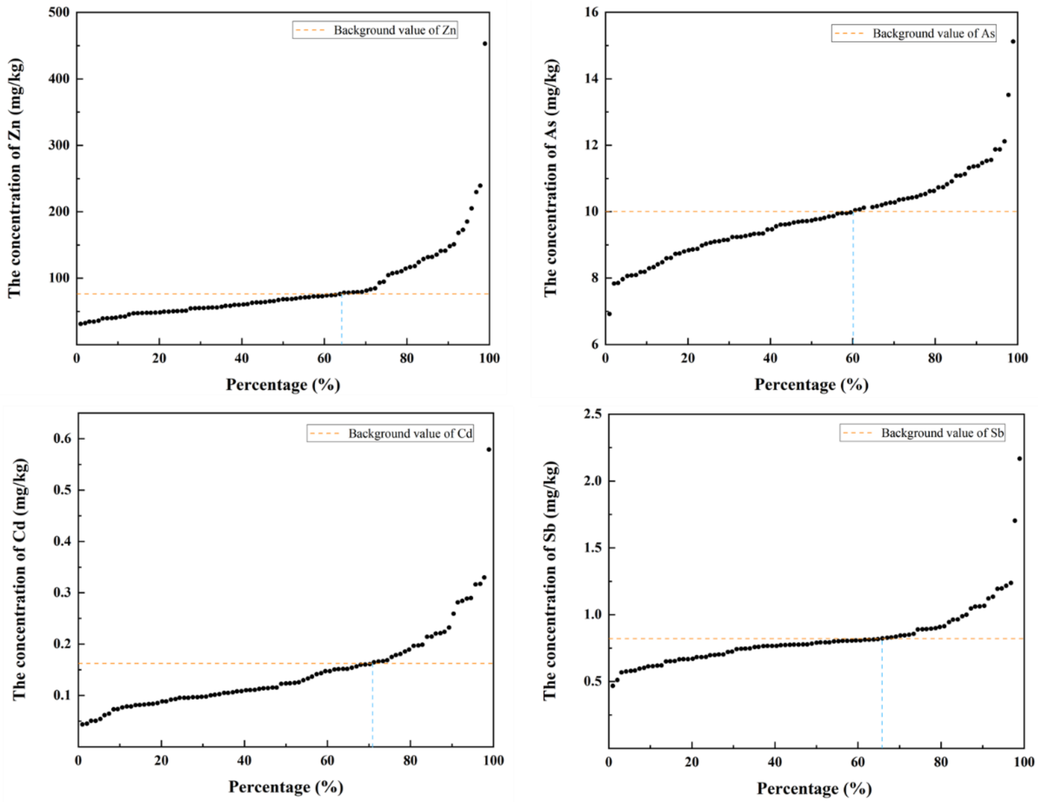

Figure S2. Cumulative probability curves of the contents of Zn, As, Cd, and Sb in soils around the complex.

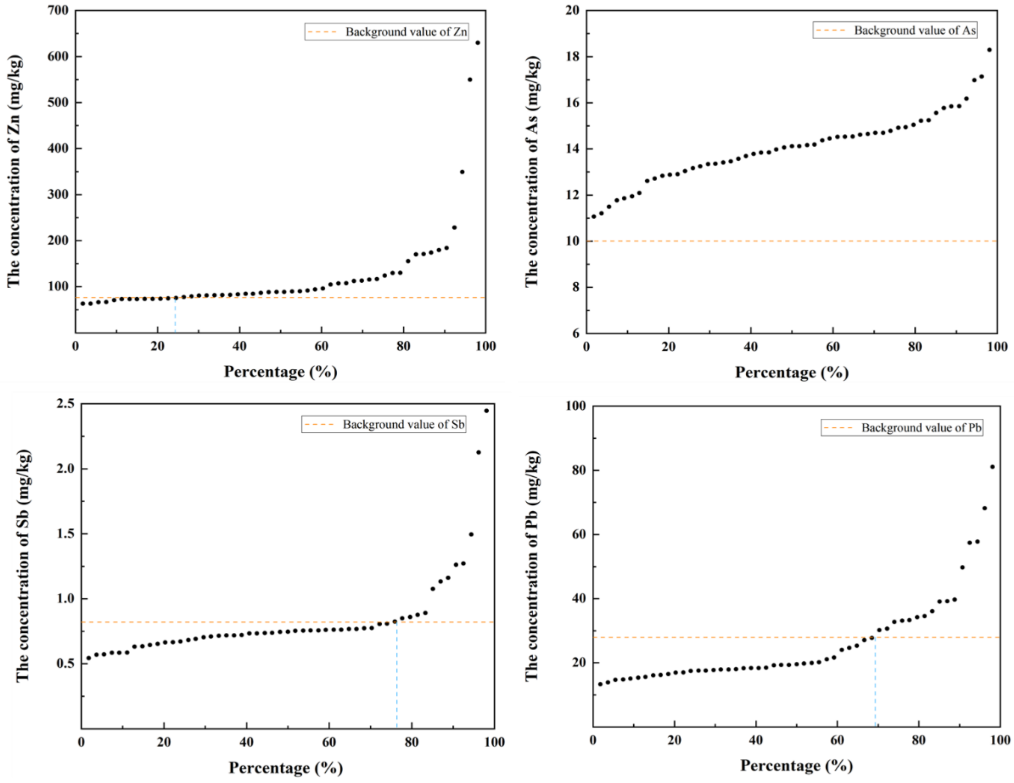

Figure S3. Cumulative probability curves of the contents of Zn, As, Sb, and Pb in soils inside the complex.

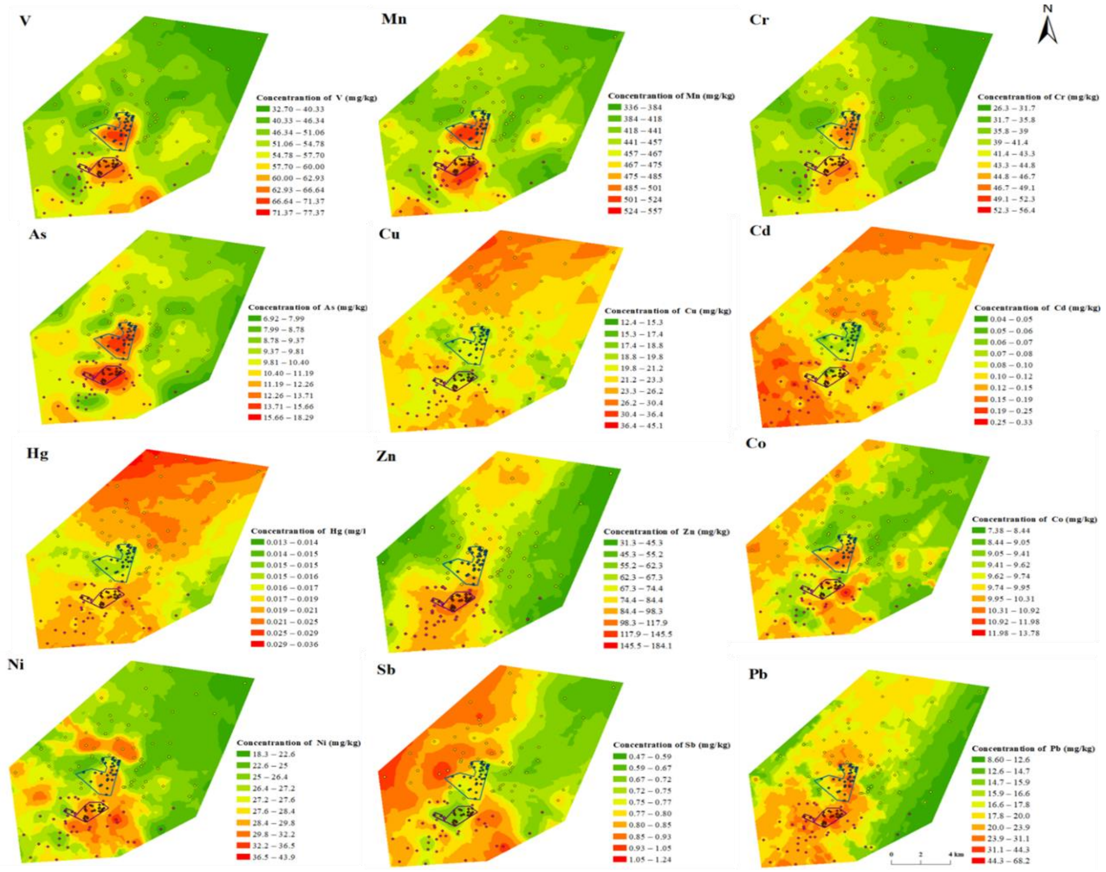

Figure S4. Estimated Ordinary Kriging concentration maps for V, Mn, Cr, As, Cu, Cd, Hg, Zn, Co, Ni, Sb, and Pb (mg/kg).

Table S1. Soil samples collection information.

Study Area	Specific Division	Sampling Area	Number of Samples
Inside the complex	South complex	Inside the south complex	24
	North complex	Inside the north complex	29
Around the complex	Around the south complex	Within 4 km around the south Complex	37
	Around the north complex	Within 10 km around the north Complex	56

Table S2. The classification of geoaccumulation index (I_{geo}).

Class	Value	Soil Quality
0	$I_{geo} \leq 0$	Uncontaminated
1	$0 < I_{geo} \leq 1$	Uncontaminated to moderately contaminated
2	$1 < I_{geo} \leq 2$	Moderately contaminated
3	$2 < I_{geo} \leq 3$	Moderately to heavily contaminated
4	$3 < I_{geo} \leq 4$	Heavily contaminated
5	$4 < I_{geo} \leq 5$	Heavily to extremely contaminated
6	$I_{geo} > 5$	Extremely contaminated

Table S3. The classification of Pollution index (*PI*) and Nemerow integrated pollution index (*NPI*).

Class	Value	Soil Quality
1	$PI/NPI < 0.7$	Safe
2	$0.7 \leq PI/NPI < 1$	Precaution
3	$1 \leq PI/NPI < 2$	Slightly contaminated
4	$2 \leq PI/NPI < 3$	Moderately contaminated
5	$PI/NPI \geq 3$	Heavily contaminated

Table S4. Parameters of human-health risk assessment models.

Factor	Description	Value		Reference
		Adults	Children	
IngR	Soil ingestion rate (mg/d)	100	200	[64]
EF	Exposure frequency (d/a)	350	350	[31]
ED	Exposure duration (a)	20	6	[31]
BW	average body weight (kg)	19.2	61.8	[64]
AT	Average time (d)	350*ED*	350*ED*	[31]
InhR	Inhalation rate (m^3/d)	14.5	7.5	[64]
PEF	particle emission factor (m^3/d)	1.36×10^9	1.36×10^9	[65]
SA	exposed skin area (cm^2)	6032	2373	[31]
AF	Skin adherence factor (mg/cm^2)	0.07	0.2	[65]
ABF	dermal adsorption factor	0.001	0.001	[32]

Table S5. Summary of reference dose (*RfD*) and slope factor (*SF*) of HMs.

Heavy Metals	*RfD* (mg/(kg·d))			*SF*((kg·d)/mg)		
	Ingestion	Inhalation	Dermal	Ingestion	Inhalation	Dermal
V	7.00×10^{-3} [b]	-	7.00×10^{-5} [b]	-	-	-
Cr	3.00×10^{-3} [a]	2.86×10^{-5} [a]	6.00×10^{-5} [a]	8.50×10^{-3} [a]	4.10×10 [c]	-
Mn	4.60×10^{-2} [b]	1.43×10^{-5} [b]	1.84×10^{-3} [b]	-	-	-
Co	2.00×10^{-2} [a]	3.00×10^{-5} [a]	1.00×10^{-2} [a]	-	9.80 [c]	-
Ni	2.00×10^{-2} [a]	2.60×10^{-2} [a]	5.40×10^{-3} [a]	-	8.40×10^{-1} [a]	-
Cu	4.00×10^{-2} [a]	4.02×10^{-2} [a]	1.20×10^{-2} [a]	-	-	-
Zn	3.00×10^{-1} [a]	3.00×10^{-1} [a]	6.00×10^{-2} [a]	-	-	-
As	3.00×10^{-4} [a]	1.23×10^{-4} [a]	1.23×10^{-4} [a]	1.50 [a]	1.51 [a]	3.66 [a]
Cd	1.00×10^{-3} [a]	1.00×10^{-5}[a]	1.00×10^{-5} [a]	6.10 [a]	6.30 [a]	-
Sb	4.00×10^{-4} [b]	-	8.00×10^{-6} [b]	-	-	-
Pb	3.50×10^{-3} [a]	3.52×10^{-3} [a]	5.25×10^{-4} [a]	8.50×10^{-3} [a]	4.20×10^{-2} [c]	-
Hg	3.00×10^{-4} [a]	8.57×10^{-5} [a]	2.10×10^{-5} [a]	-	-	-

[a] [3]; [b] [58]; [c] [59]; -, data not available.

Table S6. Concentrations (mg/kg) of HMs in soil samples in different studies.

Area	Sample	Type of data	V	Cr	Mn	Co	Ni	Cu	Zn	As	Cd	Sb	Pb	Hg	References
East China	Inside the complex	Median	63.9	45.6	494	9.77	28.4	18.7	88.4	14.1	0.072	0.75	19.5	0.016	This study
		Mean	64.7	47.1	495	9.94	31.2	19.5	207	14.0	0.10	0.84	26.2	0.019	
	Around the complex	Median	51.8	39.7	443	9.51	26.8	22.1	68.3	9.7	0.12	0.79	16.7	0.019	
		Mean	50.0	38.6	443	9.55	28.9	24.7	84.4	9.8	0.15	0.83	20.2	0.021	
Guangzhou, China	Around the complex	Median	-	-	-	-	-	17.5	146.3	4.5	0.16	-	60.4	0.45	[8]
		Mean	-	-	-	-	-	38.8	158.6	4.6	0.23	-	69.4	0.61	
Jiangsu, China	Around the complex	Median	-	49.70	-	-	23.24	44.32	53.68	6.69	0.99	-	26.69	0.43	[48]
		Mean	-	49.26	-	-	23.34	46.45	57.22	6.83	1.02	-	28.22	0.53	
Pearl River Delta, China	Natural soils	Median	-	44.7	-	2.20	8.25	7.15	36.2	-	0.39	-	28.2	-	[36]
		Mean	-	51.8	-	7.12	16.5	14.7	50.7	-	0.25	-	29.9	-	
Tarkwa, Ghana	Agricultural soils	Median	-	15	-	1.3	2.5	5.8	32	2.7	0.024	-	5.5	0.11	[35]
		Mean	-	21	-	1.8	3.7	6.2	39	4.4	0.052	-	7.2	0.32	
Northeastern Iran	Agricultural soils	Mean	-	-	5.73	-	-	1.35	0.62	-	-	-	-	-	[34]
Xinjiang, China	Petrochemical industrial city	Mean	-	117.86	-	-	42.7	64.22	123.32	20.65	0.74	-	32.56	-	[19]
Guangzhou, China	Retired industrial area	Mean	-	-	-	-	31.62	93.06	-	16.22	0.69	-	47.24	0.18	[6]
Serbia	Industrial area	Mean	43	87	430	7.2	37	110	202	6.3	0.24	-	56	35.6	[21]
	Non-industrial area	Mean	76	61	700	11.4	48	30	128	11	0.06	-	36	0.6	
	Industrial area	Mean	19.3	13.8	212.5	-	-	-	-	5.5	0.21	-	36.3	0.08	[15]

Tarragona, Spain	Residential area	Mean	13.6	10.2	191.5	-	-	-	-	4.15	0.19	-	66.1	0.08

Table S7. Descriptive statistics of HM contents (mg/kg) in soils of north complex (*n* = 24) and south complex (*n* = 29).

HMs	North					South				
	Min	Max	Median	Mean	SD	Min	Max	Median	Mean	SD
V	57.9	76.9	64.0	64.5	3.92	55.2	77.4	63.6	64.9	5.03
Cr	39.5	56.4	45.9	46.8	3.57	40.0	95.8	44.9	47.5	10.6
Mn	425	553	484	487	34.5	404	619	498	504	48.5
Co	8.47	11.8	9.90	10.1	0.77	8.48	12.8	9.68	9.79	0.96
Ni	22.3	103	28.5	33.2	16.6	23.2	49.7	28.0	28.8	5.40
Cu	13.2	34.7	19.0	20.1	4.59	14.2	30.6	17.1	18.8	4.28
Zn	63.3	4500	84.5	257	803	63.2	630	106	148	138
As	11.1	17.1	13.3	13.3	1.30	13.2	18.3	14.7	15.0	1.13
Cd	0.036	0.34	0.068	0.091	0.067	0.038	0.97	0.072	0.12	0.18
Sb	0.59	2.45	0.76	0.93	0.42	0.54	1.26	0.70	0.72	0.16
Pb	13.3	68.2	18.5	24.3	13.3	15.1	81.1	22.6	28.48	15.0
Hg	0.013	0.043	0.015	0.018	0.008	0.013	0.041	0.019	0.020	0.006

Table S8. The proportion of different soil grades of HMs inside and around the petrochemical complex classified by I_{geo}.

HMs	Inside Area		Surrounding Area	
	Uncontaminated	Uncontaminated to Moderately Contaminated	Uncontaminated	Uncontaminated to Moderately Contaminated
V	100%	0.00%	100%	0.00%
Cr	100%	0.00%	100%	0.00%
Mn	100%	0.00%	100%	0.00%
Co	100%	0.00%	100%	0.00%
Ni	100%	0.00%	100%	0.00%
Cu	100%	0.00%	98.88%	1.12%
Zn	77.08%	22.92%	85.23%	14.77%
As	79.25%	20.75%	100%	0.00%
Cd	100%	0.00%	91.30%	8.70%
Sb	100%	0.00%	98.90%	1.10%
Pb	92.31%	7.69%	100%	0.00%
Hg	100%	0.00%	100%	0.00%

Table S9. The proportion of different soil grades of HMs inside and around the petrochemical complex classified by *PI* and *NPI*.

	PI	V	Cr	Mn	Co	Ni	Cu	Zn	As	Cd	Sb	Pb	Hg	*NPI*
	Safe	3.77%	82.69%	1.96%	30.19%	2.04%	71.15%	0.00%	0.00%	90.00%	6.67%	50.00%	97.96%	0.00%
	Precaution	96.23%	17.31%	98.04%	69.81%	83.67%	21.15%	27.08%	0.00%	8.00%	82.22%	21.15%	2.04%	1.89%
Inside area	Slightly contaminated	0.00%	0.00%	0.00%	0.00%	14.29%	7.69%	60.42%	100.00%	2.00%	11.11%	23.08%	0.00%	98.11%

	Moderately contaminated	0.00%	0.00%	0.00%	0.00%	0.00%	0.00%	12.50%	0.00%	0.00%	0.00%	5.77%	0.00%	0.00%
	Heavily contaminated	0.00%	0.00%	0.00%	0.00%	0.00%	0.00%	0.00%	0.00%	0.00%	0.00%	0.00%	0.00%	0.00%
Surrounding area	Safe	82.80%	95.70%	32.26%	51.61%	10.00%	32.58%	28.41%	1.09%	44.57%	3.30%	68.54%	91.86%	1.08%
	Precaution	17.20%	4.30%	67.74%	47.31%	76.67%	55.06%	39.77%	59.78%	27.17%	63.74%	19.10%	8.14%	58.06%
	Slightly contaminated	0.00%	0.00%	0.00%	1.08%	13.33%	12.36%	29.55%	39.13%	27.17%	32.97%	12.36%	0.00%	40.86%
	Moderately contaminated	0.00%	0.00%	0.00%	0.00%	0.00%	0.00%	2.27%	0.00%	1.09%	0.00%	0.00%	0.00%	0.00%
	Heavily contaminated	0.00%	0.00%	0.00%	0.00%	0.00%	0.00%	0.00%	0.00%	0.00%	0.00%	0.00%	0.00%	0.00%

Table S10. Correlations between twelve HM concentrations in soils.

	**V**	**Cr**	**Mn**	**Co**	**Ni**	**Cu**	**Zn**	**As**	**Cd**	**Sb**	**Pb**	**Hg**
**V**	1											
**Cr**	0.87 **	1										
**Mn**	0.78 **	0.78 **	1									
**Co**	0.59 **	0.63 **	0.74 **	1								
**Ni**	0.47 **	0.65 **	0.55 **	0.59 **	1							
**Cu**	−0.26 **	0.007	−0.03	0.10	0.32 **	1						
**Zn**	0.43 **	0.54 **	0.41 **	0.17 *	0.47 **	0.22 *	1					
**As**	0.89 **	0.73 **	0.73 **	0.55 **	0.44 **	−0.32 **	0.40 **	1				
**Cd**	−0.44 **	−0.20 *	−0.20 *	−0.08	0.30 **	0.68 **	0.23 **	−0.42 **	1			
**Sb**	0.009	0.24 **	0.12	0.29 **	0.51 **	0.56 **	0.31 **	−0.07	0.59 **	1		
**Pb**	0.29 **	0.41 **	0.29 **	0.13	0.47 **	0.30 **	0.68 **	0.38 **	0.38 **	0.39 **	1	
**Hg**	−0.20 *	−0.04	−0.16	−0.17	0.24 **	0.47 **	0.20 *	−0.18 *	0.64 **	0.31 **	0.45 **	1

* Correlation is significant at the 0.05 level (2-tailed). ** Correlation is significant at the 0.01 level (2-tailed).
